# Investigation of the Lipid-Lowering Activity and Mechanism of Three Extracts from *Astragalus membranaceus*, *Hippophae rhamnoides* L., and *Taraxacum mongolicum* Hand. Mazz Based on Network Pharmacology and In Vitro and In Vivo Experiments

**DOI:** 10.3390/foods13172795

**Published:** 2024-09-02

**Authors:** Xue Yang, Mingjie Jia, Jiayuan Luo, Yuning An, Zefu Chen, Yihong Bao

**Affiliations:** 1 College of Life Sciences, Northeast Forestry University, Harbin 150040, China; 2 Key Laboratory of Forest Food Resources Utilization of Heilongjiang Province, Harbin 150040, China

**Keywords:** hyperlipidemia, network pharmacology, plant extracts, HepG2, antioxidant

## Abstract

Hyperlipidemia is a metabolic disorder characterized by abnormal lipid metabolism, resulting in lipid accumulation in the plasma. According to reports, medicinal and edible plants can reduce the risk of metabolic diseases such as hyperlipidemia. This study investigates the effects and mechanisms of *Astragalus membranaceus* extract (AME), *Hippophae rhamnoides* L. extract (HRE), and *Taraxacum mongolicum* Hand. Mazz extract (TME) on hyperlipidemia. Active compounds and potential gene targets of AME, HRE, and TME were screened using LC-MS and TCMSP databases, and hyperlipidemia targets were detected from the OMIM and DisGeNet databases. A drug-target pathway disease network was constructed through protein interactions, GO enrichment, and KEGG pathway analysis. Finally, the lipid-lowering effects of three extracts were validated through in vitro HepG2 cell and in vivo animal experiments. The results show that LC-MS and network pharmacology methodologies identified 41 compounds and 140 targets. KEGG analysis indicated that the PI3K-Akt and MAPK signaling pathways significantly treat hyperlipidemia with AHT. In vitro experiments have shown that AHT is composed of a ratio of AME:HRE:TME = 3:1:2. HepG2 cell and animal experiments revealed that AHT exhibits strong lipid-lowering and antioxidant properties, significantly regulating the levels of total cholesterol (TC), triglycerides (TG), high-density lipoprotein cholesterol (HDL-C), low-density lipoprotein cholesterol (LDL-C), superoxide dismutase (SOD), and total antioxidant capacity (T-AOC). It is worth noting that AHT can effectively downregulate the protein expression levels of p-AKT/AKT and p-PI3K/PI3K and upregulate the protein expression levels of p-AMPK/AMPK and SIRT1, verifying the results predicted by network pharmacology. This study presents a novel approach to utilizing these natural plant extracts as safe and effective treatments for hyperlipidemia.

## 1. Introduction

Hyperlipidemia (HLP) is a metabolic disorder caused by abnormal lipid metabolism or transport, resulting in one or more lipids in the plasma being higher than the normal range [1]. Its characteristics are an increase in total cholesterol (TC), triglycerides (TGs), and low-density lipoprotein cholesterol (LDL-C) levels or a decrease in circulating high-density lipoprotein cholesterol (HDL-C) levels [2]. Hyperlipidemia is recognized as a significant risk factor for nonalcoholic fatty liver disease, atherosclerosis, diabetes, and various other metabolic disorders [3]. Currently, statins are the primary treatment for hyperlipidemia. While they act quickly, long-term use may result in side effects, including a single treatment mechanism, liver and kidney complications, and muscle-related issues [4]. Consequently, developing a treatment plan with good efficacy and minimal side effects has become a research hotspot in recent years.

Medicinal and edible plants contain a variety of natural active ingredients. In comparison to traditional chemically synthesized drugs, traditional Chinese medicine, which boasts extensive experience in pharmacotherapy, is noted for its reduced adverse reactions and more pronounced long-term effects [5,6]. Previous studies have indicated that medicinal and edible plants mainly treat hyperlipidemia by improving blood lipids, antioxidation, and regulating gut microbiota [7,8,9]. *Astragalus membranaceus*, *Hippophae rhamnoides* L., and *Taraxacum mongolicum* Hand. Mazz are typical medicinal and edible plants rich in active ingredients such as flavonoids, saponins, polysaccharides, and triterpenes. They have the characteristics of lowering blood lipids, lowering blood sugar, regulating immunity, antioxidation, and being anti-inflammatory. Multiple studies have demonstrated that *Astragalus membranaceus*, *Hippophae rhamnoides* L., and *Taraxacum mongolicum* Hand. Mazz can inhibit cholesterol synthesis and regulate lipid metabolism [10,11,12]. They demonstrate significant potential in the treatment of hyperlipidemia and metabolic disorders. In recent years, due to the advancement of databases related to drugs and diseases, research in the field of bioinformatics has become increasingly popular. Network pharmacology has become widely used in studying medicinal and edible plants, which can construct networks and interactions related to drugs, targets, diseases, and pathways. Network pharmacology can conduct in-depth analysis and explore the pharmacological mechanisms of various active ingredients and targets in medicinal and edible plants [13]. Therefore, network pharmacology can be used to further elucidate the chemical composition and mechanism of the three extracts’ lipid-lowering effects.

This study initially identified the active ingredients of three extracts through liquid chromatography–mass spectrometry (LC-MS). Subsequently, network pharmacology was employed to predict the lipid-lowering mechanisms associated with these active ingredients, utilizing databases to construct an interaction network relevant to the three extracts’ treatment of hyperlipidemia. The potential lipid-lowering mechanisms of the three extracts were validated through assays conducted on HepG2 cells. Finally, a hyperlipidemia mouse model was established to assess the lipid-lowering efficacy of the three extracts. This research offers a novel strategy for the prevention and treatment of hyperlipidemia.

## 2. Materials and Methods

### 2.1. Chemicals and Reagents

*Astragalus membranaceus* extract (AME), *Hippophae rhamnoides* L. extract (HRE), and *Taraxacum mongolicum* Hand. Mazz extract (TME) were purchased from Xi’an Ruihe Biotechnology Co., Ltd. (Xi’an, China). The MTT cell proliferation and cytotoxicity assay kit and BCA protein quantification kit were purchased from Beyotime Biotechnology Co., Ltd. (Shanghai, China), while the total cholesterol (TC), triglycerides (TGs), low-density lipoprotein cholesterol (LDL-C), high-density lipoprotein cholesterol (HDL-C), aspartate aminotransferase (AST), alanine aminotransferase (ALT), superoxide dismutase (SOD), and total antioxidant capacity (T-AOC) assay kits were purchased from Nanjing Jiancheng Bioengineering Research Institute (Nanjing, China). Oleic acid and simvastatin were purchased from MACKUN Biotech (Shanghai, China).

### 2.2. LC-MS Analysis Conditions

AHT powder was measured using liquid chromatography (Vanquish, Thermo, Waltham, MA, USA) and mass spectrometry (Orbitrap Exploris 120, Thermo, Waltham, MA, USA). Firstly, AHT powder (10 mg) was dissolved in 90% methanol (3 mL) and centrifuged (10,000× *g*, 10 min, 4 °C) to obtain the supernatant. Then, 150 μL of supernatant was filtered through a 0.22 μm filter membrane into an LC vial for LC-MS analysis.

The liquid chromatography conditions were as follows: The LC analysis was performed on a Vanquish UHPLC System (Thermo Fisher Scientific, Waltham, MA, USA). A 2 μL aliquot was injected into a 2.1 × 100 mm, 1.8 µm column (Waters, Milford, MA, USA) with a flow rate of 0.3 mL/min. For LC-ESI (+)-MS analysis, the mobile phases consisted of (A) 0.1% formic acid in acetonitrile (*v*/*v*) and (B) 0.1% formic acid in water (*v*/*v*) under the following gradient: 0~1 min, 10% A; 1~5 min, 10~98% A; 5~6.5 min, 98% A; 6.5~6.6 min, 98~10% A; 6.6~8 min, 10% A. For LC-ESI (−)-MS analysis, the mobile phases consisted of (C) acetonitrile and (D) ammonium formate (5 mM) under the following gradient: 0~1 min, 10% C; 1~5 min, 10~98% C; 5~6.5 min, 98% C; 6.5~6.6 min, 98~10% C; 6.6~8 min, 10% C.

Mass spectrometric detection of metabolites was performed on Orbitrap Exploris 120 (Thermo Fisher Scientific, Waltham, MA, USA) with an ESI ion source. Simultaneous MS1 and MS/MS acquisition were used. The parameters were as follows: sheath gas pressure, 40 arb; aux gas flow, 10 arb; spray voltage, 3.50 kV and −2.50 kV for ESI (+) and ESI (−), respectively; capillary temperature, 325 °C; MS1 range, *m*/*z* 100–1000.

A quantitative list of substances was obtained using the R XCMS software (V3.12.0) package for peak detection, peak filtering, and peak alignment processing. Normalizing the total peak area was used to achieve data correction and eliminate systematic errors. The substance identification process uses the Human Metabolome Database (HMDB), LipidMaps, McCloud, and KEGG databases for retrieval and comparison. The molecular weight of metabolites was determined. The molecular formula was predicted based on the mass-to-charge ratio of parent ions in primary mass spectrometry and information on added ions, and then matched with a database. Meanwhile, fragment ions from secondary spectra were utilized for secondary qualitative identification of metabolites [14,15].

### 2.3. AHT Chemical Composition Screening and Target Prediction

From the Traditional Chinese Medicine System Pharmacology Analysis Platform (TCMSP), (http://lsp.nwu.edu.cn/tcmsp.php, accessed on 20 July 2024). The database used Huangqi, Shaji, and Pugongying as keywords, and the filtering criteria were set to OB ≥ 30% and DL ≥ 0.18. Through the UniProt database (https://sparql.uniprot.org/, accessed on 20 July 2024), all targets are standardized [16,17].

### 2.4. Prediction of Targets for Hyperlipidemia

Potential targets related to hyperlipidemia have been identified from Genecards (https://www.Genecards.org/, accessed on 22 July 2024), the DisGeNet database (https://www.disgenet.org/, accessed on 18 July 2024), and Online Mendelian Inheritance in Man (OMIM, https://www.genecards.org/, accessed on 18 July 2024). After eliminating duplicate targets, potential targets related to HLP were obtained [18,19].

### 2.5. Construction of Drug Target Network and Pathways

Firstly, in order to obtain common targets for compounds and diseases, Venny 2.1.0 was used (https://bioinfogp.cnb.csic.es/tools/venny/, accessed on 23 July 2024) to perform filtering. Common targets imported into Cytoscape 3.9.1 software to construct a drug–target disease network. The interacting targets were mapped to the STRING database (https://string-db.org/, accessed on 23 July 2024). After visualization using Cytoscape 3.9.1 software, the protein–protein interaction (PPI) network was obtained and filtered based on intermediate centrality (BC), compact centrality (CC), degree centrality (DC), eigenvector centrality (EC), local average connectivity (LAC), and network centrality (NC), with values greater than or equal to twice the median. After deleting duplicate targets, the core targets were merged to obtain 140 common targets, which were imported into the DAVID database (https://david.ncifcrf.gov/, accessed on 24 July 2024). They were used for gene ontology (GO) and Kyoto Encyclopedia of Genes and Genomes (KEGGs) analysis to explore the biological processes and signaling pathways involved in treating hyperlipidemia in AHT [20,21,22].

### 2.6. Pancreatic Lipase Inhibition Test and Combined Index Analysis

Ten mg of pancreatic lipase (PL) was accurately weighed and dissolved in 10 mL Tris HCl buffer, resulting in a 1.0 mg/mL PL solution. A volume of 8.44 μL of NPB liquid was accurately transferred, and 4 mL of acetonitrile was added to obtain a 12 mmol/L NPB solution, which was used as a substrate. The sample, PL solution, and 4-nitrophenylbutyrate were added to a 96-well plate and reacted at 37 °C for 2 h. The absorbance was measured at 405 nm [23,24]. The formula for calculating the pancreatic lipase inhibition rate is:

$$\mathrm{Inhibition}\;\mathrm{rate}\;(\%)=\frac{(A_{1}-A_{2})-(A_{3}-A_{4})}{A_{1}-A_{2}}\times 100$$

A_1_ is the absorbance value of the blank group; A_2_ is the absorbance value of the blank control group; A_3_ is the absorbance value of the sample group; and A_4_ is the absorbance value of the sample control group.

The Chou Talalay method, also known as the combination index method (CI), is widely used for quantitative evaluation of the interactions between multiple drug formulations [25]. Using the Chou and Talalay formulas, calculate the effects of each extract alone and in combination.

$$\frac{f_{a}}{f_{u}}={\left(\frac{D}{D_{m}}\right)}^{m}$$

D: Dose, D_m_: the dose that produces moderate effects, f_a_: the practical portion of the dose, m: the coefficient of the dose–response curve.

The data analysis and calculation formula for the combination drug index (CI) is as follows:

$$\mathrm{CI}=\frac{D_{1}}{D_{X1}}+\frac{D_{2}}{D_{X2}}$$

D_1_ and D_2_ are the effective concentrations when the drug combination inhibition rate is 50%, while D_X1_ and D_X2_ are effective when the drug is used alone with an inhibition rate of 50%. The CI values of the complex in each group were calculated using the combination index calculation formula and CompuSyn program to analyze the effects of the three extracts combined [26,27].

### 2.7. Cell Culture and MTT Assay

HepG2 cells were derived from the cell bank of the Chinese Academy of Sciences (Shanghai, China); DMEM was used (adding 10% fetal bovine serum and 1% penicillin and streptomycin); and the cells were cultured in a humidified incubator at 37 ℃ and 5% CO_2_, and the medium was changed every two to three days. When the cell density reached over 80%, trypsin was used for subculture [28]. HepG2 cells were detected for cell proliferation using an MTT assay. The cells were seeded at a density of 5 × 10^3^ cells/well into a 96-well plate for 24 h and then replaced with standard culture medium containing different concentrations (0, 10, 50, 100, 200, and 500 μM) of HRE, AME, TME, and AHT for 24 h. Then, 10 μL of MTT solution were added and the cells were incubated for 4 h. Afterward, 150 μL of dimethyl sulfoxide was added to each well and the absorbance value was measured at 490 nm using a microplate reader (Tecan Infinite 200 Pro, Shanghai, China) [17,29].

### 2.8. Establishment of High-Fat Model and Administration Regimen

Cells were seeded at a density of 5 × 10^5^ cells/well in a 12-well culture plate and cultured for 24 h. They were then exposed to oleic acid (0.5 mM) for 24 h to induce a high-fat model. The treatment group was cultured with 100 μM HRE, 200 μM AME, 100 μM TME, and AHT (AME: HRE: TME = 3:1:2) for 24 h.

### 2.9. Oil Red O Staining

HepG2 cells in each well were washed gently with PBS 3 times and then rinsed quickly with 60% isopropanol for 20 s. The cells were stained with Oil Red O in the dark at room temperature (25 °C) for 20 min. Then, the staining solution was discarded and the cells were rinsed 5 times with distilled water, each for 1 min per well. In addition, 100% isopropanol (1 mL) was added to each well and stirred at room temperature (25 °C) for 10 min [30].

### 2.10. Determination of Lipid-Lowering Levels in HepG2 Cells by AHT

Cells were seeded at a density of 5 × 10^5^ cells/well in a 12-well culture plate and cultured for 24 h. Except for the NC group, which was added to the standard culture medium, all other groups were added to the culture medium containing OA. The treatment group was added with AME at a concentration of 200 μM, HRE, and TME at a concentration of 100 μM, and AHT for 24 h to evaluate their lipid-lowering effects on cells. The cell culture medium was removed, and the cells were washed three times with PBS before being lysed. The levels of TC, TG, HDL-C, and LDL-C were measured according to the instructions of the reagent kit. The protein concentrations of each group were measured using the BCA protein quantification kit.

### 2.11. Western Blot Analysis

Cells were seeded at a density of 1 × 10^6^ cells/well in a 6-well culture plate and cultured for 24 h. All other groups were added to the standard culture medium except for the NC group, which was added to the culture medium containing OA. The treatment group was added with 100 μM HRE, 200 μM AME, and 100 μM TME and AHT for 24 h, respectively, and Western blotting was performed for testing. The Western blotting method is described below.

Cells were lysed in RIPA buffer (Beyotime, Shanghai, China) containing 1% PMSF for 10 min, then centrifuged at 4 °C and 12,000× *g* for 10 min. Protein quantification was performed using the BCA protein assay kit (Beyotime, Shanghai, China). The protein sample was boiled at 100 °C for 10 min and then separated by 10% SDS-PAGE. It was transferred to a PVDF Western blotting membrane (Biotopped, Beijing, China) and then blocked with 5% skim milk powder (Biosharp, Hefei, China) at room temperature (RT) for 2 h. The membrane was washed three times with TBST buffer (Biotopped, Beijing, China). The primary antibody was incubated overnight at 4 °C, and the membrane was washed three times with TBST and then incubated with the secondary antibody goat anti-rabbit (ABclonal, AS014, WB: 1:2000). An ECL chemiluminescence detection kit (ABclonal, Wuhan, China) was used for protein detection, a gel imager (Tanon 5200, Shanghai, China) was used for image capture, and image J software (Version 1.46r) was used to analyze the gray value. β—actin rabbit (ABclonal, AC038, WB: 1:20,000) was used as an endogenous control. The selected primary antibodies were: PI3 kinase p85 alpha rabbit pAb (ABclonal, A11526, WB: 1:500), AKT1 rabbit mAb (ABclonal, A17909, WB: 1:1000), phospho-AKT1-S129 rabbit pAb (ABclonal, AP1272, WB: 1:200), phospho-PI3K (ABclonal, AP0854, WB: 1:500), AMPKα1 rabbit pAb (ABclonal, AP1229, WB: 1:2000), phospho-AMPKα1 rabbit pAb (ABclonal, AP0871, WB: 1:2000), SIRT1 rabbit pAb (ABclonal, A11267, WB: 1:2000).

### 2.12. Animal Experiments

Eight-week-old male KM mice weighing 32 ± 2 g were purchased from Qingdao Petford White Mouse Breeding Professional Cooperative (Qingdao, China). The certificate number is SCXK (Lu) 2019-00003. All mice were free to eat and drink, with a 12 h/12 h light–dark cycle. All animal procedures were conducted by the National Research Council’s “Guidelines for the Care and Use of Experimental Animals” and were approved by the Ethics Committee of Northeast Forestry University (NEFU2024-011) [31].

After adaptive feeding for 1 w, KM mice were randomly divided into 6 groups based on body weight, namely normal control (NC), model control (MC), positive control (PC), low-dose administration (LD), medium-dose administration (MD), and high-dose administration (HD), with 8 mice in each group. NC was given standard feed, while the other groups were given high-fat feed for 8 w. NC and MC were given saline by gavage, the PC group was given 10 mg/kg simvastatin by gavage, and the LD group was given 41.25 mg/kg AME, 13.75 mg/kg HRE, and 27.5 mg/kg TME by gavage. MD was orally administered 82.5 mg/kg AME, 27.5 mg/kg HRE, and 55 mg/kg TME, while HD was orally administered 165 mg/kg AME, 110 mg/kg HRE, and 55 mg/kg TME for 8 w. Food intake was measured daily, and the weight of each mouse was measured weekly. After the experiment, blood was collected from the eyeballs of anesthetized mice.

### 2.13. Determination of Serum Biochemical Indicators

Mouse blood was centrifuged at 3000× *g* for 15 min to obtain mouse serum. The levels of TC, TG, LDL-C, HDL-C, AST, ALT, SOD, and T-AOC were measured in serum according to the instructions of the kit [32].

### 2.14. Statistical Analysis

All experiments were conducted in at least three parallel experiments, and the results were reported as mean ± standard deviation. Statistical analysis was conducted using GraphPad Prism 9.5, and ****, ***, **, * and ns represent *p* < 0.0001, *p* < 0.001, *p* < 0.01, *p* < 0.05, and *p* > 0.05, respectively.

## 3. Results

### 3.1. Analysis of AHT Active Ingredients

**Figure 1:**
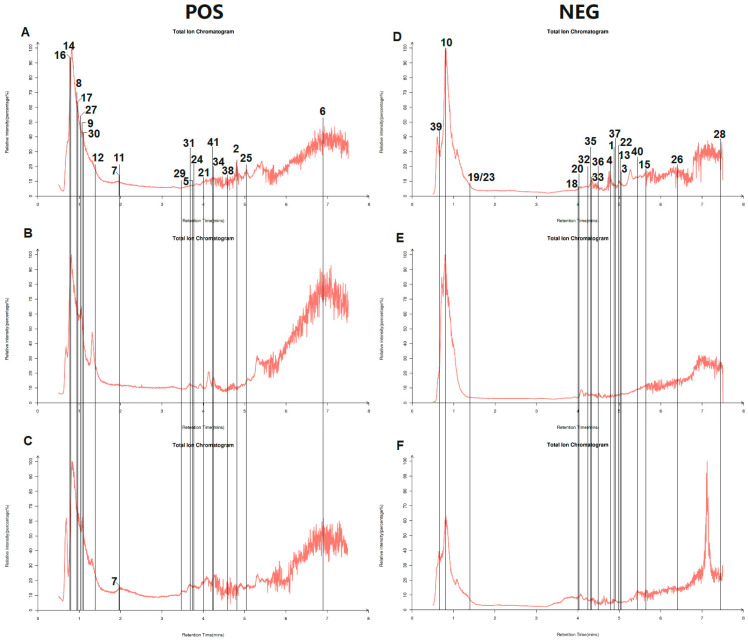
Total ion chromatogram in positive and negative ion modes. Ionic chromatograms of AME, HRE, and TME in positive-ion mode (**A**–**C**). Ionic chromatograms of AME, HRE, and TME in negative-ion mode (**D**–**F**). The numbers in Figure 1 represent the substances in Table 2.

Firstly, the active ingredients of AHT were identified, and a total of 1539 metabolites were detected by LC-MS. These metabolites include a series of compounds such as flavonoids, polyphenols, terpenes, and alkaloids. Then, 1539 compounds were identified and analyzed through the TCMSP database. Setting oral bioavailability (OB) ≥ 30% and drug-like (DL) ≥ 0.18, 41 compounds were obtained, as shown in Table 1, including bioactive ingredients such as luteolin, quercetin, isorhamnetin, and kaempferol. Next, a qualitative analysis of AHT was conducted, summarizing the retention time, *m*/*z*, molecular formula, and error (ppm) of 41 compounds (Table 2). In addition, the total ion chromatogram (TIC) of AHT in positive and negative ion modes was labeled based on the retention time of the compounds (Figure 1).

### 3.2. Potential Target Prediction of AHT and HLP

Firstly, the TCMSP database filtering criteria (OB ≥ 30%, DL ≥ 0.18) was set, a total of 256 potential targets of compounds in AHT were searched for, and all potential targets with the “Homo sapiens” species were standardized using the UniProt database to obtain Table S1. The potential targets related to hyperlipidemia were obtained from the Genecards database and Drugbank database. After eliminating duplicate targets, 1571 potential targets related to HLP were obtained (Table S2).

### 3.3. Target Network Construction for AHT and HLP

**Figure 2:**
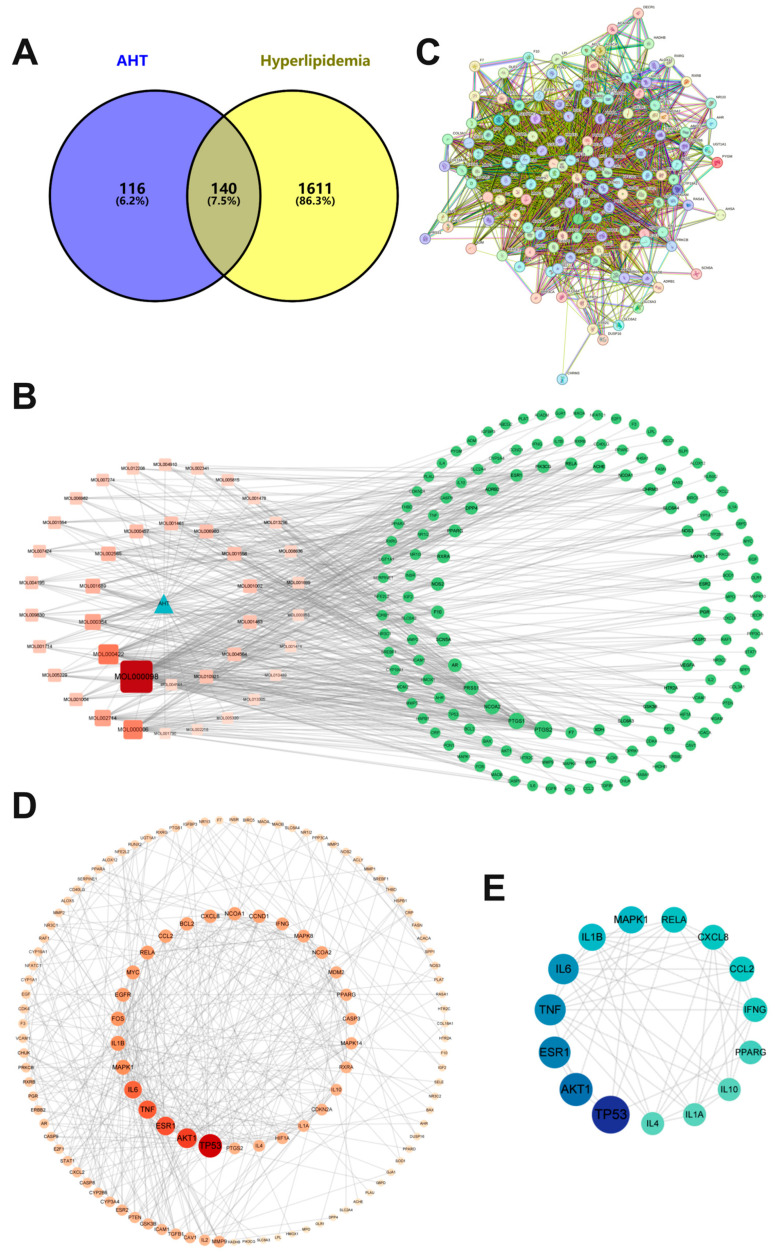
Network pharmacology analysis of AHT treatment for hyperlipidemia. (**A**) Common targets of AHT and HLP. (**B**) AHT compound target network diagram. (**C**) Analysis of protein–protein interaction networks with 140 common targets. (**D**) PPI network diagram of common targets. (**E**) Core target PPI network diagram (node size and color depth indicate the high or low DC value).

Firstly, the common targets of AHT and HLP were screened using Venny 2.1.0, resulting in 140 common targets (Figure 2A, Table S3). These targets are potential targets for AHT treatment of HLP. The above targets were imported into Cytoscape 3.9.1 software to construct a drug–target disease network. There are 181 nodes and 546 edges in Figure 2B, and the size of the nodes in the figure is related to the degree centralities (DCs) value. The 40 compounds of AHT are represented by red squares; green circles represent common targets; and blue triangles represent AHT. The results show that quercetin, kaempferol, luteolin, baicalein, isorhamnetin, acacetin, medicarpin, phaseollidin, papaverine, and dihydrochelerythrine ranked in the top ten in terms of DC values, suggesting that they are key compounds.

### 3.4. Construction of PPI Network

Common targets were imported into the STRING 11.0 database for PPI network analysis (Figure 2C). There were a total of 139 nodes and 395 edges, with an average node degree of 5.68. Import the cross targets obtained through STRING database analysis into Cytoscape 3.9.1 software to obtain the PPI network diagram for AHT treatment of HLP. As shown in Figure 2D, the larger the degree value, the darker the color and the larger the area of the gene target, indicating that the target has a more significant influence in the PPI network. In addition, the target’s BC, CC, DC, EC, LAC, and NC values were obtained through network topology analysis in Cytoscape 3.9.1 software. By screening and removing duplicate targets from the first six targets in each item, 15 key targets were obtained (Figure 2E and Table 3), namely TP53, PPARG, ESR1, TNF, CCL2, AKT1, RELA, MAPK1, IL6, CXCL8, IL1A, IL4, IL10, IL1B, and IFNG. We speculate that they may be key targets for treating HPL.

### 3.5. Enrichment Analysis of GO and KEGG Pathways

**Figure 3:**
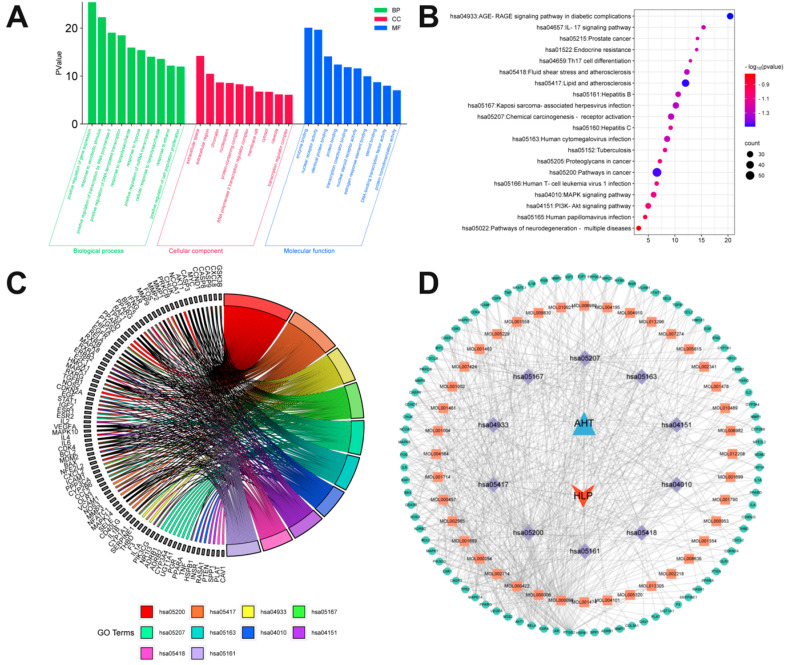
Network pharmacology pathway analysis of AHT treatment for HLP. (**A**) GO enrichment analysis. (**B**) The top 20 KEGG enrichment pathways. (**C**) KEGG chord diagram, it indicated the top 10 pathways and their corresponding targets; the different colors of the graphics represent different signal pathways. (**D**) Compound–target–pathway–disease network diagram.

To explore the potential therapeutic mechanism of AHT for hyperlipidemia we imported the overlapping genes of AHT and HLP into the DAVID database for GO and KEGG pathway enrichment analysis. Firstly, visual analysis was conducted on biological progress (BP), cellular components (CC), and molecular functions (MF). the top 10 enriched GO terms were identified separately (*p*-Value < 0.05) and the key targets were analyzed (Figure 3A). The results show that in BP, the targets of AHT were associated with response to lipolysis (GO: 0032496) and cellular response to lipolysis (GO: 0071222). In MF, the target of AHT was associated with steroid binding (GO: 0005496). In CC, the targets of AHT were associated with membrane raft (GO: 0045121), protein-containing complex (GO: 0032991), and transcription regulator complex (GO: 0005667). The GO enrichment analysis results indicated that compounds in AHT can exert therapeutic effects on hyperlipidemia from lipopolysaccharides, inflammatory factors, and protein regulation. The false discovery rate (FDR) was used as the X-axis; the size of the dots represented the number of targets in different pathways, and dots of different colors represented the p-values of different pathways. The darker the red color, the higher the significance. As shown in Figure 3B, enrichment analysis was performed on the top 20 signaling pathways for AHT treatment of hyperlipidemia. It was found that AHT mainly occurs through lipids and atherosclerosis, the AGE–RAGE signaling pathway in diabetic complications, and the inflammatory pathway, MAPK signaling pathway, and PI3K-Akt signaling pathway play a role. Table 4 presents detailed information on the top 10 signaling pathways and their corresponding targets. Moreover, the target and signaling pathway were visualized in Figure 3C. The above 10 pathways connected with relevant key targets and compounds to form a compound–target–signaling pathway network. It included 140 nodes and 657 edges (Figure 3D). A significant correlation was indicated between key signaling pathways and the main compounds of AHT. The core compounds included quercetin (MOL000098, edge count = 67), luteolin (MOL000006, edge count = 31), kaempferol (MOL000422, edge count = 27), and baicalein (MOL002714, edge count = 18).

### 3.6. Determination of AHT Compounding Ratio

An orthogonal experiment was conducted to determine the optimal blending ratio of AME, HRE, and TME. According to the theoretical analysis of the k value, the optimal combination is A_3_H_1_T_2_, which meant the ratio of AME, HRE, and TME was 3:1:2. To verify this ratio, the pancreatic lipase inhibition rate was 97.74 ± 0.62% (Table 5).

### 3.7. Combination Index Analysis of AHT, AME, HRE, and TME

**Figure 4:**
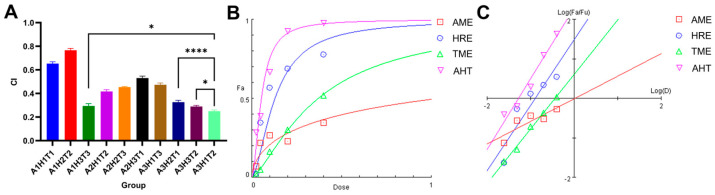
Combination Index analysis of AHT, AME, HRE, and TME. (**A**) CI values are combined in different proportions. (**B**) Dose effect curves of AHT, AME, HRE, and TME on pancreatic lipase inhibition. (**C**) Intermediate effect diagrams of AHT, AME, HRE, and TME. (*n* = 3, **** and * represent *p* < 0.01 and *p* < 0.05, respectively).

To further determine whether the combination of various extraction solutions has a synergistic effect, the dose–response relationship between each individual extraction solution and the combination group was studied, and the combination index (CI) value of each experimental group was calculated based on the principle of medium effect. The CI value of less than 1 indicated a synergistic effect between the components. As shown in Figure 4A, the CI values of all groups were less than 1, indicating that the combination of the three extracts showed sound synergistic effects. The CI value of AHT (A_3_H_1_T_2_) was significantly lower than that of the other groups (*p* < 0.05), indicating that AHT had the best synergistic effect. Figure 4B showed that the pancreatic lipase inhibition effect of AHT was superior to that of AME, HRE, and TME at various addition ratios. Figure 4C illustrates that when the synergistic effect reached 50%, the drug activity size was AHT > HRE > TME > AME. The above results indicated that the in vitro lipid-lowering effect of AHT was due to the combination of a single extract and other ratios.

### 3.8. The Effects of AHT, AME, HRE, and TME on HepG2 Cell Proliferation and Oleic Acid Staining

**Figure 5:**
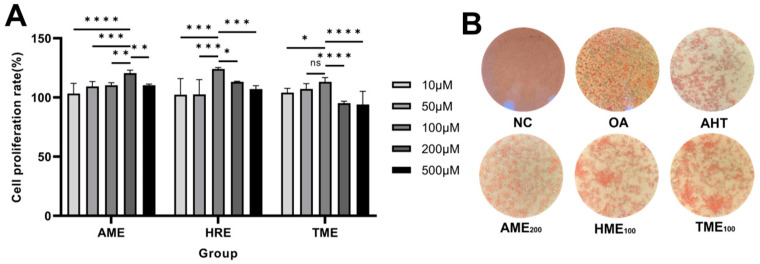
Effects of AHT, AME, HRE, and TME on HepG2 cell proliferation and oleic acid staining. (**A**) Cytotoxicity assay. (**B**) Oleic acid staining image. (*n* = 3, ****, ***, **, *, and ns represent *p* < 0.0001, *p* < 0.001, *p* < 0.01, *p* < 0.05, and *p* > 0.05, respectively).

To verify the lipid-lowering effect of AHT, we chose to establish a high-fat blood model using HepG2 cells. Before establishing the model, the cytotoxicity of AME, HRE, and TME were first detected by MTT assay. As shown in Figure 5A, AME and HRE had a promoting effect on cell proliferation at concentrations of 200 μM and 100 μM, while TME had a significant promoting effect at concentrations of 50 μM and 100 μM (*p* > 0.05). TME at a concentration of 100 μM was selected for the next experiment. When the dosage continued to increase, the activity of cells was inhibited. Next, the optimal concentrations of AME, HRE, and TME were compounded in a ratio of 3:1:2 to obtain the compound extract AHT. The lipid-lowering effect of AHT was verified. HepG2 cells were induced to produce lipid droplets using 0.5 mM OA, and the lipid droplets inside the cells were observed by staining with Oil Red O. Figure 5B showed that no obvious lipid droplet staining was observed in the NC group, and the cell morphology was good. Red lipid droplets were clearly observed within the OA group cells, covering the entire cell periphery. After adding AHT, AME, HRE, and TME, a decrease in intercellular lipid droplets was observed. The results showed that AHT, AME, HRE, and TME could alleviate lipid deposition in HepG2 cells to varying degrees.

### 3.9. The Lipid-Lowering Effect of AHT on Oleic Acid-Induced HepG2 Cells

**Figure 6:**
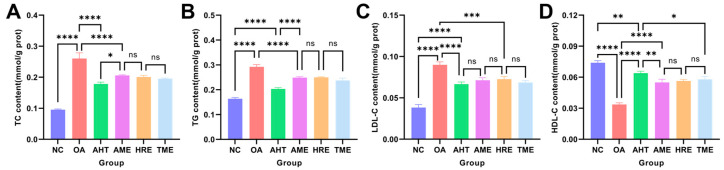
Determination of AHT lipid-lowering levels. (**A**) TC. (**B**) TG. (**C**) LDL-C. (**D**) HDL-C. (*n* = 3, ****, ***, **, *, and ns represent *p* < 0.0001, *p* < 0.001, *p* < 0.01, *p* < 0.05, and *p* > 0.05, respectively).

The HepG2 cell model induced by oleic acid (OA) had been widely used for in vitro studies of hyperlipidemia (Figure 6). Compared with the NC group, HepG2 cells induced by OA showed significantly increased levels of TC, TG, and LDL-C (*p* < 0.01) and significantly decreased levels of HDL-C (*p* < 0.01). After administration, compared with the OA group, the levels of AHT, AME, HRE, and TME decreased by 31.53%, 21.04%, 22.95%, and 24.88% (*p* < 0.01) in TC, 30.37%, 14.84%, 14.50%, and 18.84% (*p* < 0.01) in TG, 25.93%, 20.74%, 19.26%, and 23.70% (*p* < 0.01) in LDL-C, and 90.10%, 63.37%, 67.33%, and 72.28% (*p* < 0.01) in HDL-C, respectively. It indicated that AHT, AME, HRE, and TME all have good lipid-lowering effects, and the lipid-lowering performance of AHT after compounding has been further improved.

### 3.10. The Effect of AHT on the Lipid-Lowering Mechanism Induced by Oleic Acid in HepG2 Cells

**Figure 7:**
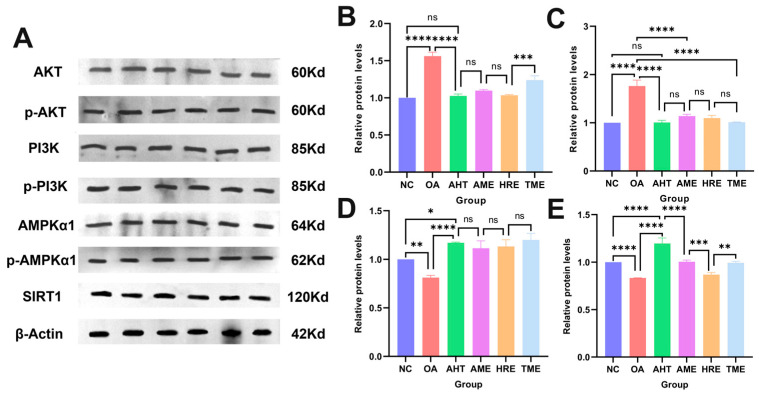
The effect of AHT on critical proteins in the PI3K Akt signaling pathway and MAPK signaling pathway. (**A**) Immunoblot analysis of related proteins. (**B**) The ratio of p-AKT/AKT protein quantification results. (**C**) The ratio of p-PI3K/PI3K protein quantification results. (**D**) The ratio of p-AMPK/AMPK protein quantification results. (**E**) SIRT1 protein quantification results. (*n* = 3, ****, ***, **, *, and ns represent *p* < 0.0001, *p* < 0.001, *p* < 0.01, *p* < 0.05, and *p* > 0.05, respectively).

Based on the prediction of lipid-lowering-related proteins by network pharmacology, we treated HepG2 cells induced by oleic acid with AHT, AME, HRE, and TME and verified the effects of the MAPK signaling pathway and the PI3K-Akt signaling pathway on the lipid-lowering mechanism of HepG2 cells (Figure 7A). As shown in Figure 7B,C, the protein expression of AKT, phosphorylated AKT (p-AKT), PI3K, and phosphorylated PI3K (p-PI3K) were validated and the relevant protein ratios were calculated. It was found that the protein expression levels of p-AKT/AKT and p-PI3K/PI3K in HepG2 cells induced by oleic acid were significantly reduced after treatment with AHT, AME, HRE, and TME (*p* < 0.05). AHT significantly reduced the phosphorylation of AKT and PI3K in HepG2 cells (*p* < 0.01). As shown in Figure 7D,E, AHT significantly increased the protein expression levels of AMPKα1, phosphorylated AMPKα1, and SIRT1 (*p* < 0.05). The above results indicated that AHT could regulate lipid function and improve lipid metabolism balance through the MAPK signaling pathway and the PI3K-Akt signaling pathway.

### 3.11. The Effect of AHT on Body Weight, Food Intake, and Organ Index in Mice

**Figure 8:**
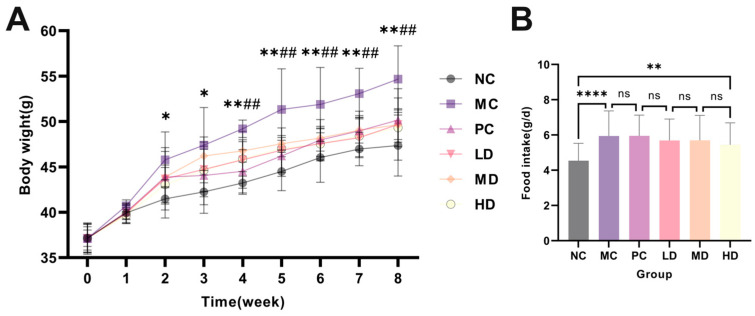
Body weight and food intake of mice. (**A**) Changes in body weight of mice in each group during 8 w. (*n* = 8, *, ** represent significant and extremely significant differences between the MC group and the NC group (*p* < 0.05, *p* < 0.01); ## represents extremely significant differences between the MC group and the HD group (*p* < 0.05). (**B**) During the 8 w period, the food intake of mice in each group. (*n* = 3, ****, **, and ns represent *p* < 0.0001, *p* < 0.01, and *p* > 0.05, respectively).

As shown in Figure 8A, there was no significant difference in the initial body weight of mice at 0 w (*p* > 0.05). With the prolongation of time, at 2 w, the weight of the MC group fed with a high-fat diet was significantly higher than that of the NC group fed with a normal diet (*p* < 0.05). At 4 w, the weight of the MC group was significantly higher than that of the HD group (*p* < 0.01). However, as shown in Figure 8B, there was no significant difference in food intake among the groups fed a high-fat diet (*p* > 0.05). It indicated that a high-fat diet could lead to weight gain in mice, and when AHT was supplemented, it prevented weight gain induced by a high-fat diet. But there was no significant difference in organ index, indicating that AHT had no adverse effects on mice (*p* > 0.05) (Table 6).

### 3.12. The Effect of AHT on Serum Biochemical Indicators in High-Fat Mice

**Figure 9:**
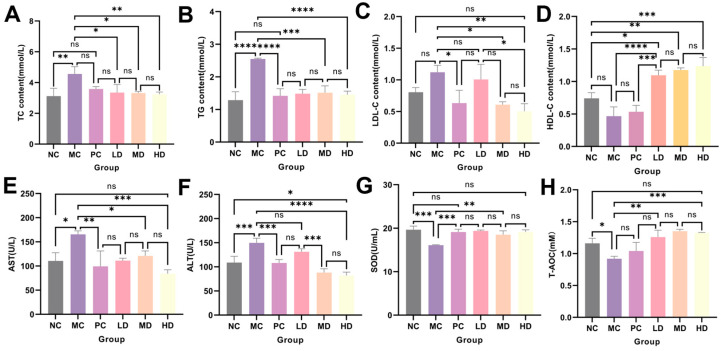
Serum indicators. (**A**) TC. (**B**) TG. (**C**) LDL-C. (**D**) HDL-C. (**E**) AST. (**F**) ALT. (**G**) SOD. (**H**) T-AOC. (*n* = 3, ****, ***, **, *, and ns represent *p* < 0.0001, *p* < 0.001, *p* < 0.01, *p* < 0.05, and *p* > 0.05, respectively).

Blood lipids, liver damage, and antioxidant levels in mouse serum were measured. As shown in Figure 9A–D, compared with the NC group, the levels of TC, TG, and LDL-C were significantly increased in the MC group, while the level of HDL-C was significantly decreased. The LD group, MD group, and HD group all showed varying degrees of improvement, with TC, TG, and LDL-C levels in the HD group decreasing by 29.01%, 42.98%, and 55.01%, respectively, compared to the MC group (*p* < 0.01), while HDL-C levels increased significantly by 165.85% (*p* < 0.0001). AST and ALT are considered indicators for detecting liver injury and can verify the damage of hyperlipidemia to the liver of mice. As shown in Figure 9E,F, the AST and ALT levels in the HD group decreased by 49.19% and 45.23%, respectively, compared to the MC group and returned to normal levels. Dysregulation of lipid metabolism could lead to the production of large amounts of reactive oxygen species in the body, causing oxidative stress. Therefore, we validated the antioxidant level and found that the antioxidant capacity of the MC group significantly decreased, while the SOD and T-AOC levels of the HD group significantly increased by 18.99% and 44.20%, respectively, compared to the MC group (*p* < 0.01) (Figure 9G,H).

## 4. Discussion

Long-term high-fat diets will lead to a disorder of lipid metabolism and increase the risk of chronic diseases such as obesity, hyperlipidemia, and diabetes [33]. Hyperlipidemia is a complex disease; the traditional intervention method is taking statins. However, these drugs usually have a single site of action, and long-term use can cause some toxic side effects. Previous studies have found that disturbances in liver lipid metabolism, oxidative stress, and chronic inflammation in the body often accompany the occurrence of hyperlipidemia [34]. Therefore, using natural plant ingredients with multi-target, multi-pathway, natural, safe, and efficient characteristics to prevent or intervene in lipid metabolism disorders has attracted widespread attention from researchers [35]. *Astragalus membranaceus*, *Hippophae rhamnoides* L., and *Taraxacum mongolicum* Hand. Mazz are traditional edible and medicinal plants with a long history of consumption. They have been widely reported to have various bioactive functions, such as lowering blood lipids, antioxidation, and immune regulation [36,37,38]. Therefore, this study aimed to explore the lipid-lowering effects, targets, and mechanisms of the combined application of *Astragalus membranaceus* extract, *Hippophae rhamnoides* extract, and dandelion extract.

We first identified the main compounds in *Astragalus membranaceus* extract, *Hippophae rhamnoides* L. extract, and *Taraxacum mongolicum* Hand. Mazz extract using LC-MS and identified 1539 compounds. Research has confirmed that compound plants have better regulatory effects on lipid metabolism/diseases than single plants. Previous studies have shown that AME, HRE, and TME all have lipid-lowering activity, but their potential mechanisms for preventing hyperlipidemia have yet to be fully explored. Further research is needed to verify their mechanisms of action, such as key compounds, targets, and mechanisms. Therefore, network pharmacology was used to predict the targets and signaling pathways of the combined intervention of AME, HRE, and TME in hyperlipidemia. A total of 41 compounds and 140 potential targets for treating hyperlipidemia were detected. Fifteen core targets, including TP53, PPARG, ESR1, AKT1, RELA, and MAPK1, were identified through PPI analysis. Key compounds such as quercetin, luteolin, kaempferol, and baicalein were identified through compound target pathway analysis. These key compounds and core targets may be key factors in treating hyperlipidemia. Research has shown that the main component of the polyphenol-rich extract from *Allium cepa* and *Gynostemma pentaphyllum* is quercetin, demonstrating that quercetin can achieve therapeutic effects on hyperlipidemia through LOX1-PI3K AKT eNOS [39,40]. In addition, *Astragalus membranaceus* can regulate lipid metabolism by downregulating AKT1 and upregulating ESR1 [41]. These results are similar to our research findings [42]. The results of KEGG enrichment analysis indicate that AHT may regulate hyperlipidemia through lipids and atherosclerosis, MAPK signaling pathway, and PI3K-Akt signaling pathway.

To verify the predictive results of network pharmacology, AME, HRE, and TME were combined to obtain AHT. The lipid-lowering ability, antioxidant capacity, and mechanism of action of AHT were validated by establishing oleic acid-induced HepG2 cell models and high-fat diet-induced hyperlipidemia mouse models. The results showed that AHT can regulate lipid function, alleviate oxidative stress, and have an excellent lipid-lowering effect through the MAPK and PI3K-Akt signaling pathways. Research shows that the PI3K-Akt signaling pathway can participate in atherosclerosis activity and improve glucose and lipid metabolism in mice [43,44]. In this study, AHT can regulate the PI3K-Akt signaling pathway and activate the expression of SIRT1 protein. SIRT1, as a dependent acylase, is involved in lipid metabolism disorders such as hyperlipidemia, which reduces the protein expression level of SITR1 [45]. The SIRT1/AMPK pathway can regulate lipid content, increase mitochondrial fatty acid oxidation, and promote lipid breakdown and energy metabolism. In Zheng et al.’s study [46], SDF-PPs effectively reversed the decrease in p-AMPK/AMPK and inhibited the activity of acetyl CoA carboxylase 1 (ACC1). Our research also found that AMPK phosphorylation was enhanced in HepG2 cells treated with AHT. Previous reports have shown that α-ketoglutarate (AKG) activates AMPK protein in liver cells in dyslipidemia, enhancing its phosphorylation. After AMPK phosphorylation enhancement, it can inhibit the increase in TC and TG and oxidative stress induced by palmitic acid in HepG2 cells [47]. Our results also found a significant decrease in TC, TG, and LDL-C levels in cells after AHT intervention, as well as a significant increase in HDL-C levels. It indicates that AHT regulates the lipid breakdown ability in liver cells by activating the MAPK and PI3K-Akt signaling pathways, inhibiting TC and TG accumulation in HepG2 cells and mice. The above results indicate that AHT can enhance the regulatory ability of liver cells toward lipid metabolism and their resistance to oxidative stress. To further evaluate the lipid-lowering effect of AHT, we established a high-fat mouse model using a high-fat diet. After 8 weeks of AHT intervention, we found that the levels of TC, TG, and LDL-C in the HD group were significantly reduced, HDL-C levels were significantly increased, AST and ALT activities were decreased, and SOD and T-AOC levels were increased [7,48,49]. These results indicate that AHT can improve lipid metabolism disorders in high-fat mice by increasing the body’s antioxidant levels and reducing liver damage. In summary, we validated the targets and pathways predicted by network pharmacology through cell and animal experiments. We found that AHT can improve hyperlipidemia and regulate lipid metabolism by regulating the MAPK and PI3K-Akt signaling pathways.

## 5. Conclusions

In this work, network pharmacology was used to predict and analyze the potential mechanisms of bioactive compounds in AME, HRE, and TME for treating hyperlipidemia, and the lipid-lowering mechanism of AHT was explored through in vitro and in vivo experiments. The research results indicate that compounds in AHT, such as quercetin, luteolin, kaempferol, and baicalein, might affect hyperlipidemia by targeting core targets such as TP53, PPARG, ESR1, AKT1, RELA, MAPK1, as well as by acting on the PI3K-Akt and MAPK signaling pathways. In vitro and in vivo experiments showed that AHT could reduce TC, TG, and LDL-C levels, increase HDL-C levels, reduce liver injury, and enhance the body’s antioxidant capacity. Most importantly, AHT could improve hyperlipidemia by affecting the expression of proteins related to the PI3K-Akt and MAPK signaling pathways. In summary, these findings provide a new strategy for AHT to serve as a safe and efficient resource for improving lipid metabolism disorders such as hyperlipidemia.

## Figures and Tables

**Table 1:** Active ingredients of AME, HRE, and TME.

No.	Name	Molecule ID	Molecule Name	OB (%)	DL
1	Luteolin	MOL000006	Luteolin	36.16	0.25
2	Quercetin	MOL000098	Quercetin	46.43	0.28
3	Isorhamnetin	MOL000354	Isorhamnetin	49.6	0.31
4	Kaempferol	MOL000422	Kaempferol	41.88	0.24
5	Phaseollidin	MOL000457	Phaseollidin	52.04	0.53
6	Cholesterol	MOL000953	CLR	37.87	0.68
7	Ellagic acid	MOL001002	Ellagic acid	43.06	0.43
8	Pelargonidin	MOL001004	Pelargonidin	37.99	0.21
9	Dihydrochelerythrine	MOL001461	Dihydrochelerythrine	32.73	0.81
10	Dihydrosanguinarine	MOL001463	Dihydrosanguinarine	59.31	0.86
11	Sanguinarine	MOL001474	Sanguinarine	37.81	0.86
12	Chelerythrine	MOL001478	Toddaline	25.99	0.81
13	Scopolamine	MOL001554	Scopolamine	67.97	0.27
14	(+)-Sesamin	MOL001558	Sesamin	56.55	0.83
15	Acacetin	MOL001689	Acacetin	34.97	0.24
16	Diphyllin	MOL001699	Diphyllin	36.23	0.75
17	Podofilox	MOL001714	Podophyllotoxin	59.94	0.86
18	Linarin	MOL001790	Linarin	39.84	0.71
19	Scopolin	MOL002218	Scopolin	56.45	0.39
20	Hesperetin	MOL002341	Hesperetin	70.31	0.27
21	Medicarpin	MOL002565	Medicarpin	49.22	0.34
22	Baicalein	MOL002714	Baicalein	33.52	0.21
23	Melilotoside	MOL004101	Melilotoside	36.85	0.26
24	Corydaline	MOL004195	CORYDALINE	65.84	0.68
25	Kaempferide	MOL004564	Kaempferid	73.41	0.27
26	Glabranin	MOL004910	Glabranin	52.9	0.31
27	Artemetin	MOL005229	Artemetin	49.55	0.48
28	Arachidonic acid	MOL005320	Arachidonate	45.57	0.2
29	Citromitin	MOL005815	Citromitin	86.9	0.51
30	Papaverine	MOL006980	Papaverine	64.04	0.38
31	Codeine	MOL006982	Codeine	45.48	0.56
32	Cirsimaritin	MOL007274	Skrofulein	30.35	0.3
33	Artemisinin	MOL007424	Artemisinin	49.88	0.31
34	Corynoline	MOL008636	Corynoline	30.53	0.85
35	Camptothecin	MOL009830	EHD	61.04	0.81
36	Leucocyanidin	MOL010489	Resivit	30.84	0.27
37	Estrone	MOL010921	Estrone	53.56	0.32
38	Lobelanine	MOL012208	Lobelanine	54.13	0.32
39	Uridine 5’-monophosphate	MOL012820	5’-Uridylic acid	40.25	0.2
40	Fustin	MOL013296	Fustin	50.91	0.24
41	Garbanzol	MOL013305	Garbanzol	83.67	0.21

**Table 2:** The compound composition in AHT determined by LC-MS.

Peak No.	Proposed Compound	RT/s	Precursor *m*/*z*	Error (ppm)	Formula
1	Luteolin	290	285.0761	2.606	C_15_H_10_O_6_
2	Quercetin	287	303.0498	0.420	C_15_H_10_O_7_
3	Isorhamnetin	303.7	315.0515	1.512	C_16_H_12_O_7_
4	Kaempferol	282	285.0391	4.770	C_15_H_10_O_6_
5	Phaseollidin	224	325.1379	17.003	C_20_H_20_O_4_
6	CLR	412.7	369.3588	19.607	C_27_H_46_O
7	Ellagic acid	116.6	324.9999	13.574	C_14_H_6_O_8_
8	Pelargonidin	57.5	543.1263	4.175	C_15_H_11_O_5_
9	Dihydrochelerythrine	63.8	332.1285	1.169	C_21_H_19_NO_4_
10	Dihydrosanguinarine	48.1	665.1907	3.352	C_20_H_15_NO_4_
11	Sanguinarine	116.9	289.1103	1.990	C_20_H_14_NO_4_
12	Toddaline	83.9	332.1315	10.197	C_21_H_18_NO_4_
13	Scopolamine	301.9	285.1163	10.784	C_17_H_21_NO_4_
14	Sesamin	48.5	337.1115	13.218	C_20_H_18_O_6_
15	Acacetin	336.1	283.0606	2.098	C_16_H_12_O_5_
16	Diphyllin	46.8	381.0936	8.594	C_21_H_16_O_7_
17	Podophyllotoxin	58.8	829.2688	1.687	C_22_H_22_O_8_
18	Linarin	240.2	653.184	17.888	C_28_H_32_O_14_
19	Scopolin	81.8	335.0758	4.287	C_16_H_18_O_9_
20	Hesperetin	241.6	301.071	2.518	C_16_H_14_O_6_
21	Medicarpin	240.9	254.0728	11.289	C_16_H_14_O_4_
22	Baicalein	297.6	269.0448	2.767	C_15_H_10_O_5_
23	Melilotoside	81.8	371.0968	4.221	C_15_H_18_O_8_
24	CORYDALINE	226.1	387.2221	14.781	C_22_H_27_NO_4_
25	Kaempferid	302.1	299.0567	1.977	C_16_H_12_O_6_
26	Glabranin	385.4	369.1471	4.907	C_20_H_20_O_4_
27	Artemetin	61.3	345.1367	9.972	C_20_H_20_O_8_
28	Arachidonate	447.9	303.2351	7.121	C_20_H_32_O_2_
29	Citromitin	206.6	405.1339	18.738	C_21_H_24_O_8_
30	Papaverine	66.5	322.127	4.678	C_20_H_21_NO_4_
31	Codeine	219.3	300.1587	2.372	C_18_H_21_NO_3_
32	Skrofulein	252	359.073	11.803	C_17_H_14_O_6_
33	Artemisinin	260.9	343.1382	4.764	C_15_H_22_O_5_
34	Corynoline	254.3	350.1458	3.569	C_21_H_21_NO_5_
35	EHD	260.3	330.2363	11.189	C_20_H_1_6N_2_O_4_
36	Resivit	267.9	288.0443	14.498	C_15_H_14_O_7_
37	Estrone	292.4	269.079	10.881	C_18_H_22_O_2_
38	Lobelanine	276	336.1978	5.963	C_22_H_25_NO_2_
39	5’-Uridylic acid	41.1	323.0274	3.682	C_9_H_13_N_2_O_9_P
40	Fustin	326.2	333.0473	15.279	C_15_H_12_O_6_
41	Garbanzol	252.4	273.0805	17.407	C_15_H_12_O_5_

**Table 3:** Prediction of key targets for AHT treatment of HPL.

NO.	Symbol ID	Protein Name	Pathways
1	TP53	Tumor protein p53	hsa05417, hsa04010, hsa04151
2	PPARG	Peroxisome proliferator-activated receptor gamma	hsa05417
3	ESR1	Estrogen receptor	hsa05207
4	TNF	Tumor necrosis factor	hsa05417, hsa04933, hsa04010
5	CCL2	C-C motif chemokine 2	hsa05417, hsa04933, hsa05418
6	AKT1	Potassium channel AKT1	hsa05417, hsa04933, hsa04010, hsa04151
7	RELA	Transcription factor p65	hsa05417, hsa04933, hsa04010, hsa04151
8	MAPK1	Mitogen-activated protein kinase 1	hsa05417, hsa04933, hsa04010, hsa04151
9	IL6	Interleukin-6	hsa05417, hsa04933, hsa04151
10	CXCL8	Interleukin-8	hsa05417, hsa04933
11	IL1A	Interleukin-1 alpha	hsa04933, hsa04010, hsa05418
12	IL4	Interleukin-4	hsa04151
13	IL10	Interleukin-10	-
14	IL1B	Interleukin-1 beta	hsa05417, hsa04933, hsa04010
15	IFNG	Interferon gamma	hsa05418

**Table 4:** Annotation of the top 10 KEGG pathways.

ID	Description	*p*-Value	Gene ID	Count
hsa05200	Pathways in cancer	29.15	GSK3B, CXCL8, PTEN, CASP9, CASP8, CCND1, MYC, CASP3, AKT1, NCOA1, CHUK, PRKCB, MMP1, MMP2, FOS, MMP9, AR, IFNG, BIRC5, PPARG, RAF1, TP53, PPARD, PTGS2, HIF1A, EGFR, RELA, RXRB, MAPK8, RXRA, ERBB2, E2F1, HMOX1, MAPK1, RXRG, TGFB1, NOS2, CDKN2A, EGF, STAT1, IGF2, ESR1, ESR2, IL2, VEGFA, MAPK10, IL4, IL6, CDK4, BCL2, MDM2, BAX, NFE2L2	53
hsa05417	Lipid and atherosclerosis	30.27	GSK3B, CXCL8, TNF, CXCL2, RELA, ICAM1, CASP9, RXRB, PPP3CA, MAPK8, CASP8, CYP2B6, RXRA, CASP3, CCL2, AKT1, MAPK1, OLR1, RXRG, VCAM1, CHUK, MMP1, NOS3, MMP3, NFATC1, FOS, MAPK14, SELE, MMP9, MAPK10, IL6, CD40LG, IL1B, CYP1A1, BCL2, BAX, PPARG, TP53, NFE2L2	39
hsa04933	AGE-RAGE signaling pathway in diabetic complications	31.05	CXCL8, SERPINE1, TNF, RELA, ICAM1, THBD, MAPK8, CCND1, CASP3, CCL2, AKT1, MAPK1, TGFB1, VCAM1, PRKCB, NOS3, STAT1, MMP2, NFATC1, MAPK14, SELE, F3, VEGFA, MAPK10, IL1A, COL3A1, IL6, CDK4, IL1B, BCL2, BAX	31
hsa05167	Kaposi sarcoma-associated herpesvirus infection	20.65	GSK3B, CXCL8, PTGS2, HIF1A, CXCL2, RELA, PIK3CG, ICAM1, CASP9, PPP3CA, MAPK8, CASP8, CCND1, MYC, CASP3, E2F1, AKT1, MAPK1, CHUK, STAT1, NFATC1, FOS, MAPK14, VEGFA, MAPK10, IL6, CDK4, BAX, RAF1, TP53	30
hsa05207	Chemical carcinogenesis—receptor activation	19.50	NR1I3, ADRB1, AHR, ADRB2, CYP3A4, RELA, EGFR, RXRB, CYP2B6, RXRA, CCND1, MYC, E2F1, AKT1, MAPK1, RXRG, UGT1A1, PRKCB, EGF, FOS, ESR1, ESR2, VEGFA, AR, CYP1A1, BCL2, BIRC5, PGR, RAF1, PPARA	30
hsa05163	Human cytomegalovirus infection	17.81	GSK3B, CXCL8, PTGS2, TNF, RELA, EGFR, CASP9, PPP3CA, CASP8, CCND1, MYC, CASP3, E2F1, CCL2, AKT1, MAPK1, CHUK, CDKN2A, PRKCB, NFATC1, MAPK14, VEGFA, IL6, CDK4, IL1B, MDM2, BAX, RAF1, TP53	29
hsa04010	MAPK signaling pathway	12.69	HSPB1, TNF, RELA, EGFR, PPP3CA, MAPK8, MYC, CASP3, ERBB2, AKT1, MAPK1, TGFB1, CHUK, PRKCB, EGF, INSR, IGF2, NFATC1, FOS, MAPK14, VEGFA, MAPK10, IL1A, RASA1, IL1B, RAF1, TP53	27
hsa04151	PI3K-Akt signaling pathway	10.81	GSK3B, PTEN, RELA, EGFR, PIK3CG, CASP9, RXRA, CCND1, MYC, ERBB2, SPP1, AKT1, MAPK1, CHUK, EGF, NOS3, INSR, IGF2, IL2, VEGFA, IL4, IL6, CDK4, BCL2, MDM2, RAF1, TP53	27
hsa05418	Fluid shear stress and atherosclerosis	19.75	PLAT, TNF, RELA, ICAM1, THBD, MAPK8, CCL2, AKT1, HMOX1, VCAM1, CHUK, NOS3, CAV1, MMP2, FOS, MAPK14, SELE, MMP9, VEGFA, MAPK10, IL1A, IFNG, IL1B, BCL2, TP53, NFE2L2	26
hsa05161	Hepatitis B	18.15	CXCL8, TNF, RELA, CASP9, MAPK8, CASP8, MYC, CASP3, E2F1, AKT1, MAPK1, TGFB1, CHUK, PRKCB, STAT1, NFATC1, FOS, MAPK14, MMP9, MAPK10, IL6, BCL2, BAX, BIRC5, RAF1, TP53	26

**Table 5:** Pancreatic lipase inhibition rates of AME, HRE, and TME with different compounding ratios.

Group	AME	HRE	TME	Inhibition Rate%
1	1	1	1	80.71 ± 0.83
2	1	2	2	79.84 ± 0.80
3	1	3	3	72.11 ± 1.94
4	2	1	2	94.25 ± 1.10
5	2	2	3	86.08 ± 0.86
6	2	3	1	89.64 ± 1.23
7	3	1	3	96.72 ± 1.30
8	3	2	1	95.87 ± 0.25
9	3	3	2	96.47 ± 0.32
k_1_	77.55	90.56	88.74	
k_2_	89.99	87.26	90.19	
k_3_	96.35	86.07	84.97	
R	18.80	4.49	5.13	
Best combination	A_3_H_1_T_2_			

**Table 6:** The effect of AHT on organ indices in different groups of mice.

Group	NC	MC	PC	LD	MD	HD
Heart (%)	0.693 ± 0.11	0.672 ± 0.11	0.602 ± 0.10	0.707 ± 0.19	0.660 ± 0.09	0.613 ± 0.10
Liver (%)	4.554 ± 0.47	4.527 ± 0.35	3.847 ± 1.40	4.750 ± 0.67	4.483 ± 0.66	4.548 ± 0.74
Spleen (%)	0.327 ± 0.03	0.309 ± 0.05	0.253 ± 0.06	0.306 ± 0.08	0.282 ± 0.10	0.321 ± 0.09
Kidney (%)	1.462 ± 0.18	1.367 ± 0.13	1.357 ± 0.17	1.463 ± 0.19	1.334 ± 0.23	1.271 ± 0.14

(*n* = 8, *p* > 0.05; there was no significant difference between the groups).

## Data Availability

The original contributions presented in the study are included in the article and Supplementary Materials, further inquiries can be directed to the corresponding author.

## Supplementary Materials

The following supporting information can be downloaded at: https://www.mdpi.com/article/10.3390/foods13172795/s1, Table S1: Drug targets, Table S2: Target of hyperlipidemia, Table S3: Targets of drugs and diseases.

## List of Abbreviations

ALT	Alanine aminotransferase
AST	Aspartate aminotransferase
AME	*Astragalus membranaceus* extract
AHT	*Astragalus membranaceus* extract: *Hippophae rhamnoides* L. extract: *Taraxacum mongolicum* Hand. Mazz extract = 3:1:2
HDL-C	High-density lipoprotein cholesterol
HRE	*Hippophae rhamnoides* L. extract
LC-MS	Liquid chromatography–mass spectrometry
LDL-C	Low-density lipoprotein cholesterol
OMIM	Online Mendelian Inheritance in Man
PPI	Protein–protein interaction
SOD	Superoxide dismutase
TME	*Taraxacum mongolicum* Hand. Mazz extract
TCMSP	Traditional Chinese Medicine System Pharmacology Analysis Platform
T-AOC	Total antioxidant capacity
TC	Total cholesterol
TG	Triglycerides
